# Effect of the sphingosine kinase 1 selective inhibitor, PF-543 on arterial and cardiac remodelling in a hypoxic model of pulmonary arterial hypertension

**DOI:** 10.1016/j.cellsig.2016.03.014

**Published:** 2016-08

**Authors:** Neil MacRitchie, Giora Volpert, Mohammed Al Washih, David G. Watson, Anthony H. Futerman, Simon Kennedy, Susan Pyne, Nigel J. Pyne

**Affiliations:** a Strathclyde Institute of Pharmacy and Biomedical Science, University of Strathclyde, Glasgow G4 0RE, UK; b Department of Biological Chemistry, Weizmann Insitute of Science, Rehovot 76100, Israel; c Institute of Cardiovascular and Medical Sciences, College of Medical, Veterinary & Life Sciences, University of Glasgow, G12 8QQ, UK

**Keywords:** Sphingosine kinase, Sphingosine 1-phosphate, Hypoxia, Pulmonary arterial hypertension, Hypertrophy, Vascular remodelling

## Abstract

Recent studies have demonstrated that the expression of sphingosine kinase 1, the enzyme that catalyses formation of the bioactive lipid, sphingosine 1-phosphate, is increased in lungs from patients with pulmonary arterial hypertension. In addition, *Sk1*^−/−^ mice are protected from hypoxic-induced pulmonary arterial hypertension. Therefore, we assessed the effect of the sphingosine kinase 1 selective inhibitor, PF-543 and a sphingosine kinase 1/ceramide synthase inhibitor, RB-005 on pulmonary and cardiac remodelling in a mouse hypoxic model of pulmonary arterial hypertension. Administration of the potent sphingosine kinase 1 inhibitor, PF-543 in a mouse hypoxic model of pulmonary hypertension had no effect on vascular remodelling but reduced right ventricular hypertrophy. The latter was associated with a significant reduction in cardiomyocyte death. The protection involves a reduction in the expression of p53 (that promotes cardiomyocyte death) and an increase in the expression of anti-oxidant nuclear factor (erythroid-derived 2)-like 2 (Nrf-2). In contrast, RB-005 lacked effects on right ventricular hypertrophy, suggesting that sphingosine kinase 1 inhibition might be nullified by concurrent inhibition of ceramide synthase. Therefore, our findings with PF-543 suggest an important role for sphingosine kinase 1 in the development of hypertrophy in pulmonary arterial hypertension.

## Highlights

• PF-543, a sphingosine kinase 1 inhibitor reduces cardiac hypertrophy in a mouse pulmonary arterial hypertension (PAH) model
• This results in reduced cardiomyocyte apoptosis
• PF-543 reduces PARP processing and p53 expression and increases Nrf-2 expression in the right ventricle of mice with PAH

## 1 Introduction

Sphingosine 1-phosphate (S1P) is formed by the phosphorylation of sphingosine, catalysed by sphingosine kinase isoforms (SK1 and SK2), which are encoded by distinct genes and differ in their subcellular localisation, biochemical properties and functions. The S1P formed by these enzymes can either be exported from cells (through transporter proteins *e.g.* Spns2) and bind to a family of five S1P-specific G protein coupled receptors (S1P_1–5_) [1], [2] or can interact with specific intracellular target proteins. For instance S1P formed by nuclear SK2 inhibits HDAC1/2 activity to induce c-fos and p21 expression [3]. Dephosphorylation of S1P is catalysed by S1P phosphatase and the sphingosine formed is then acylated to ceramide, catalysed by ceramide synthase isoforms [4]. S1P can also be irreversibly cleaved by S1P lyase to produce (*E*)-2 hexedecenal and phosphoethanolamine [5].

Pulmonary arterial hypertension (PAH) is a debilitating progressive lung disease primarily affecting small pre-capillary arteries within the lung vasculature and is associated with vasoconstriction, vascular remodelling and pro-inflammatory changes. Current treatments for PAH are targeted at improving pulmonary haemodynamics, exercise tolerance and quality of life. Currently licenced drugs for treating PAH are all vasodilators of three main classes: prostacyclin analogues, endothelin receptor antagonists and phosphodiesterase 5 inhibitors. The introduction of vasodilator therapy has markedly improved life expectancy from less than 3 years at diagnosis in the pre-treatment era to 5–7 years with current optimal combination therapy [6]. However, they fail to halt the progressive vascular and ventricular remodelling that ultimately determines mortality amongst PAH patients. Hypoxia, which induces PAH, is associated with increased expression of SK1 in human pulmonary arterial smooth muscle cells (PASMCs) [7] and this might, therefore, be a contributing factor to the vascular remodelling in PAH. Indeed, SK1 expression is increased in lungs from patients with PAH and *Sk*1^−/−^ mice are protected from hypoxic-induced pulmonary hypertension [8]. The S1P formed by SK1 binds to the S1P_2_ receptor to promote vascular smooth muscle proliferation [8]. A variety of cardiovascular diseases such as PAH lead to a compensatory adaptive increase in cardiac muscle mass *e.g.* hypertrophy. However, this results in dysfunctional hypertrophy which eventually progresses to right ventricular failure (RVF), the primary cause of death in pulmonary hypertensive patients. Indeed, short-term in hospital mortality for patients admitted with PAH associated RVF vary from 14–41% [9], [10], [11], [12]. The pathophysiological mechanisms that result in RVF are still unclear. It is well documented that apoptosis is a key feature of left ventricular failure [13], [14], [15] and recent evidence also implicates apoptosis as an important mechanism in RVF. Serial imaging of apoptosis in right ventricular dysfunction shows apoptosis increasing as right ventricular function declines [16] and pharmacologically induced reductions in right ventricular hypertrophy (RVH) are associated with reduced apoptosis [17].

Preventing apoptosis of cardiomyocytes, therefore, is a major objective for the treatment of RVH. In this regard, deletion of the *Sk2* gene in mice is associated with a considerable increase in ischaemic reperfusion-induced injury and a reduction in the cardio-protective effect of ischaemic preconditioning [18]. These findings suggest that SK2 exerts a beneficial function against heart failure. Recent studies have also shown that S1P levels are markedly increased after myocardial infarction (MI) and this is associated with elevated expression of SK1 and S1P_1_ in the heart [19]. Moreover, inhibition of SK1 with the nM potent SK1 selective inhibitor, PF-543 reduced post-MI cardiac remodelling and dysfunction [19]. Indeed, inhibition of S1P lyase to increase S1P levels enhanced cardiac remodelling and dysfunction. S1P was shown to enhance β1-adrenergic receptor stimulation-induced pro-inflammatory responses in the cardiomyocytes and FTY720 [19], a functional S1P_1_ antagonist [20] and SK1 inhibitor [21] reduced cardiac SK1/S1P/S1P_1_ signalling, ameliorated chronic cardiac inflammation and cardiac remodelling and dysfunction *in vivo* post-MI [19].

Given the potential role of SK1 in cardiac and vascular remodelling, we have assessed the effect of the selective nM potent SK1 inhibitor, PF-543 on pulmonary and cardiac remodelling in a mouse hypoxic model of PAH. PF-543 exhibits a K_i_ for inhibition of SK1 activity of 14 nM and inhibits SK2 by only 33% at 5 μM [22]. We have compared PF-543 with a modestly potent inhibitor of SK1, RB-005 (IC_50_ = 3.6 μM for SK1 inhibition [23]), which we show here also inhibits ceramide synthase (CerS). Thus, the rationale for comparing PF-543 with RB-005 is as follows. Inhibition of SK1 by PF-543 is likely to promote accumulation of sphingosine which can be back converted to ceramide by ceramide synthase. Similarly, inhibition of SK1 by RB-005 is likely to induce accumulation of sphingosine, but the inhibition of ceramide synthase by RB-005 might be expected to prevent back conversion to ceramide. Therefore comparative effects of PF-543 and RB-005 might be instructive in delineating the impact of ceramide biosynthesis on PAH.

In this study, we assessed the effect of the SK1 selective inhibitor, PF-543 and a SK1/ceramide synthase inhibitor, RB-005 on pulmonary and cardiac remodelling in a mouse hypoxic model of PAH. Our findings suggest that SK1 might play an important role in the development of dysfunctional hypertrophy in PAH.

## 2 Material and methods

### 2.1 Animals

C57BL/6 mice were purchased from Harlan (Oxford, UK) and maintained at the Biological Procedure Unit, University of Strathclyde. All experiments were performed under the guidelines of the UK Home Office Animals (Scientific Procedures) Act 1986 and were compliant with the ARRIVE guidelines for experiments involving animals [24]. Female mice at the age of 7–12 weeks were used in all experiments. These were then randomly divided them into separate cages and each cage randomly designated to a specific treatment (Cage A = PF-543, Cage B = RB-005 and Cage C = vehicle) in a non-blinded manner.

### 2.2 Pharmacokinetics

2 month old female mice (C57BL/6 J) (Harlan, Oxford, UK) were injected *via* the tail vein with RB-005 or PF-543 (10 or 30 mg/kg) dissolved in vehicle (20% (2-Hydroxypropyl)-β-cyclodextrin in phosphate buffered saline (PBS)). 20 μL blood was withdrawn *via* tail vein bleeds at 15 min, 30 min, 1 h, 4 h, 6 h and 24 h following drug administration. All experiments were performed under the guidelines of the UK Home Office Animals (Scientific Procedures) Act 1986 and were compliant with the ARRIVE guidelines for experiments involving animals [24]. Drug concentration was determined by MS analysis.

### 2.3 MS analysis

Analysis of the levels of RB-005 and PF-543 in blood was carried out by using an Agilent 6460 triple quadrupole instrument (Agilent, Stockport, UK) operated at + 4 kV with a sheath gas flow of 7 L/min and a gas temperature of 250 °C. The MS was interfaced with a 1260 infinity quaternary HPLC pump fitted with an ACE C18 column (150 × 3 mm, 3 μm particle size, HiChrom Reading UK). The mobile phase was acetonitrile/water (50:50) containing 0.1% *v*/v formic acid with a flow rate of 0.5 ml/min. Chlorpromazine (20 ng) added to each blood sample as an internal standard and then an equal volume of acetonitrile was added and protein was removed by centrifugation. The following transitions were monitored in MRM mode chlorpromazine (319 → 86, 319 → 58, collision energy (CE) 30 V), PF-543 (466 → 448, 466 → 223, CE 30 V), RB-005 (318 → 217, 318 → 119 CE 30 V). Calibration curves were constructed by adding standards in the range 0–200 ng to blood samples from non-treated animals. Plotting the ratio of internal standard response over responses for the analytes resulted in calibration curves with R^2^ > 0.99.

### 2.4 Hypoxic induced PAH

Mice were placed into a hypobaric chamber and maintained at 550 mbar atmospheric pressure (~ 10% O_2_ concentration) for a period of 3 weeks. Every second day mice received an intraperitoneal injection of RB-005, PF-543 (10 mg/kg and 1 mg/kg, respectively) or vehicle (20% (2-Hydroxypropyl)-β-cyclodextrin in PBS). The chamber was depressurized and then repressurised in order to inject the mice [25].

### 2.5 Measurement of right ventricular pressure

Anaesthesia of mice was induced by 3% isoflurane in O_2_ and body weight measured. Anaesthesia was maintained by 1–2% isoflurane in O_2_ supplied *via* a face mask. Right ventricular pressure (RVP) was measured by transdiaphragmatic cardiac puncture utilizing a 25G hypodermic needle attached to a fluid filled pressure transducer (ADinstruments, Oxford, UK) which was advanced through the diaphragm and into the right ventricle. Mice were euthanized by cervical dislocation and the heart and lungs dissected for subsequent analysis.

### 2.6 Measurement of right ventricular hypertrophy

Atria and large calibre blood vessels were dissected from the rest of the heart. The right ventricle (RV) free wall was then dissected away from the left ventricle (LV) and septum (S). The LV + S along with the RV were blotted dry and weighed separately. The ratio of RV/(LV + S) was used as an index of RVH.

### 2.7 Assessment of vascular remodelling

Sagittal sections from the right lung (5 μm) were fixed, paraffin embedded and stained with Elastica-Van Gieson stain (Merck Millipore, Nottingham, UK) before being microscopically assessed for evidence of muscularisation of small pulmonary arteries (< 80 μm diameter). Arteries were considered muscularised if they possessed a distinct double-elastic lamina visible around at least 70% of the vessel circumference. At least 150 vessels per section were counted. The percentage of muscularised (remodelled) vessels was calculated as the number of remodelled vessels / total number of vessels counted × 100.

### 2.8 Assessment of ventricular apoptosis

Right ventricular tissue sections (5 μm) were fixed, paraffin embedded and DNA strand breaks detected by a Terminal deoxynucleotidyl transferase (TdT) dUTP Nick-End Labelling (TUNEL) assay kit (Roche, Burgess Hill, UK) according to the manufacturer's instructions. Sections were then counterstained with haematoxylin and apoptotic cells were automatically counted using ImageJ (NIH) software. At least 400 cells were counted per heart and apoptotic index calculated as number of apoptotic cells / total number of cells counted × 100.

### 2.9 Cell culture

Human PASMC were grown in human smooth muscle cell growth medium supplemented with growth supplement, 100 U/mL penicillin and 100 μg/mL streptomycin at 37 °C in 5% CO_2_.

### 2.10 Western blotting

PASMC extracts for SDS-PAGE and Western blot analysis were prepared by washing treated cells with 5 ml of PBS and then re-suspending cell pellets in whole cell lysis buffer [(137 mM NaCl, 2.7 mM KCl, 1 mM MgCl_2_, 1 mM CaCl_2_, 1% *v*/v NP40, 10% *v*/v glycerol, 20 mM Tris) (pH 8.0) containing 0.2 mM PMSF, 10 μg/mL leupeptin, 10 μg/mL aprotinin, 0.5 mM Na_3_VO_4_, 100 μM NaF, and 10 mM β-glycerophosphate]. Samples were repeatedly (× 6) passed through a 23 gauge needle using a syringe and rotated for 60 min at 4 °C to allow for efficient lysis. Cell debris was pelleted by centrifugation at 22,000 *g* for 10 min at 4 °C and the supernatant (whole cell extract) was collected. Right ventricular tissue was placed into modified RIPA buffer [(50 mM Tris, 150 mM NaCl, 1 mM EDTA, 1% Triton X-100, 0.5% sodium deoxycholate, 0.1% SDS) (pH 7.5) containing 0.2 mM PMSF, 10 μg/mL leupeptin, 10 μg/mL aprotinin, 0.5 mM Na_3_VO_4_, 100 μM NaF, and 10 mM β-glycerophosphate] before being pulverized using a liquid N_2_ cooled mortar and pestle maintained on dry ice. Samples were rotated for 60 min at 4 °C before being centrifuged at 22,000 g for 10 min at 4 °C. Supernatants were then collected. The protein content of PASMCs and ventricular lysates was measured using the Pierce BCA Assay Kit (Fisher Scientific UK, Loughborough). For each sample, 10–20 μg of protein, combined with Lamelli buffer [(0.5 mM Tris, 2 mM Na_4_P_2_O_7_, 5 mM EDTA, 2% *w*/*v* SDS) (pH 6.7) containing 12.5% *v*/v glycerol, 0.25% *w*/*v* bromophenol blue, and 50 mM dithiothreitol] was used for SDS-PAGE and Western blotting. Proteins were separated on a 10% (*v*/v) acrylamide/bisacrylamide gel, and transferred to a nitrocellulose Hybond membrane (GE Healthcare, Little Chalfont, UK). Membranes were blocked in 5% (*w*/*v*) bovine serum albumin (BSA) (Fisher Scientific UK, Loughborough) in TBST buffer (20 mM Tris HCl (pH 7.5), 48 mM NaCl, 0.1% (*v*/v) Tween20) for 1 h at room temperature prior to incubation with primary antibody (diluted in blocking buffer) overnight at 4 °C. Following three washes in TBST, membranes were incubated with horse radish peroxidase-conjugated anti-mouse or anti-rabbit IgG secondary antibody (Sigma, Poole, UK; diluted in blocking buffer), as appropriate, for 1 h at room temperature. Immunoreactive protein bands were visualized using enhanced chemiluminescence. Primary antibodies were sourced as follows: phospho-p38 MAPK (#9211), phospho-ERK2 (#9101), phospho-STAT3 (#9131), ERK2 (#9108), PARP (#2965) from Cell Signalling Technologies (NEB, Hitchin, UK), PCNA (sc-56) and Nrf2 (sc-13032) from Santa Cruz (Insight Biotechnology, Wembley, UK), actin (A2547), p53 (P8999) and fibronectin (F3648) from Sigma (Poole, UK). Anti-SK1 (lab reference number 48:2) antibody was custom made by Abcam using antigens detailed in [26].

### 2.11 Sphingosine kinase activity assays

Right ventricular homogenate was incubated for 60 min at 30 °C, in the presence of 3 μM sphingosine, 250 μM [γ-^32^P]ATP (37 kBq) in 20 mM Tris (pH 7.4), 1 mM EDTA, 1 mM Na_3_VO_4_, 40 mM β-glycerophosphate, 1 mM NaF and 500 μM MgCl_2_ in the presence of Triton X-100 (~ 0.6% *v*/v). Assay reactions were terminated by the addition of 500 μL of 1-butanol. After 1 mL of 2 M KCl was added, with mixing, two phases were formed. The lower (aqueous) phase, which contains unreacted [γ-^32^P]ATP, was removed and discarded. The organic phase containing [^32^P]-S1P was extracted by washing twice with 2 M KCl (1 mL each time) before quantification by Cerenkov counting.

### 2.12 CerS assay

Cell homogenates from HEK 293 cells overexpressing CerS5 were incubated at 37 °C with 15 μM NBD-Sph (Avanti Polar Lipids, Alabaster, USA), 20 μM defatted BSA and 50 μM acyl CoA in a 20 μl reaction volume according to Tidhar et al., [27] Reactions were terminated by the addition of chloroform/methanol (1:2, *v*/v) and lipids were extracted. Lipids were then dried under N_2_, resuspended in chloroform/methanol (9:1, *v*/v) and separated by TLC using chloroform/methanol/2 M NH_4_OH (40:10:1, *v*/v/v) as the developing solvent. NBD-labelled lipids were visualized using a Typhoon 9410 variable mode imager and quantified by ImageQuantTL (GE Healthcare, Chalfont St Giles, UK).

### 2.13 Caspase-3/7 assay

PASMCs (10,000 per well) were seeded into white walled 96 well plates and allowed to adhere for 24 h. Subsequently, media was replenished with 100 μL of media containing either PF-543 (100 nM or 1 μM), RB-005 (1 or 10 μM), vehicle control or positive control (proteasomal inhibitor, MG132; 10 μM) and incubated for a further 2 to 4 h. Caspase-Glo® 3/7 Reagent (Promega, Southampton, UK) was prepared according to the manufacturer's instructions and 100 μL was added to each well and incubated at room temperature for 2 h before reading on a luminometer. Following caspase cleavage, a substrate for luciferase (aminoluciferin) is released, resulting in the luciferase reaction and the production of light. The luminescent signal intensity produced is proportional to the amount of caspase 3/7 activity. Luminescent signal was acquired using a Wallac Victor plate reader (Perkin Elmer Seer Green, UK).

## 3 Results and discussion

### 3.1 Effect of PF-543 and RB-005 on body weight, blood concentration, SK1 activity and expression in pulmonary arteries and cultured human pulmonary arterial smooth muscle cells

**Fig. 1:**
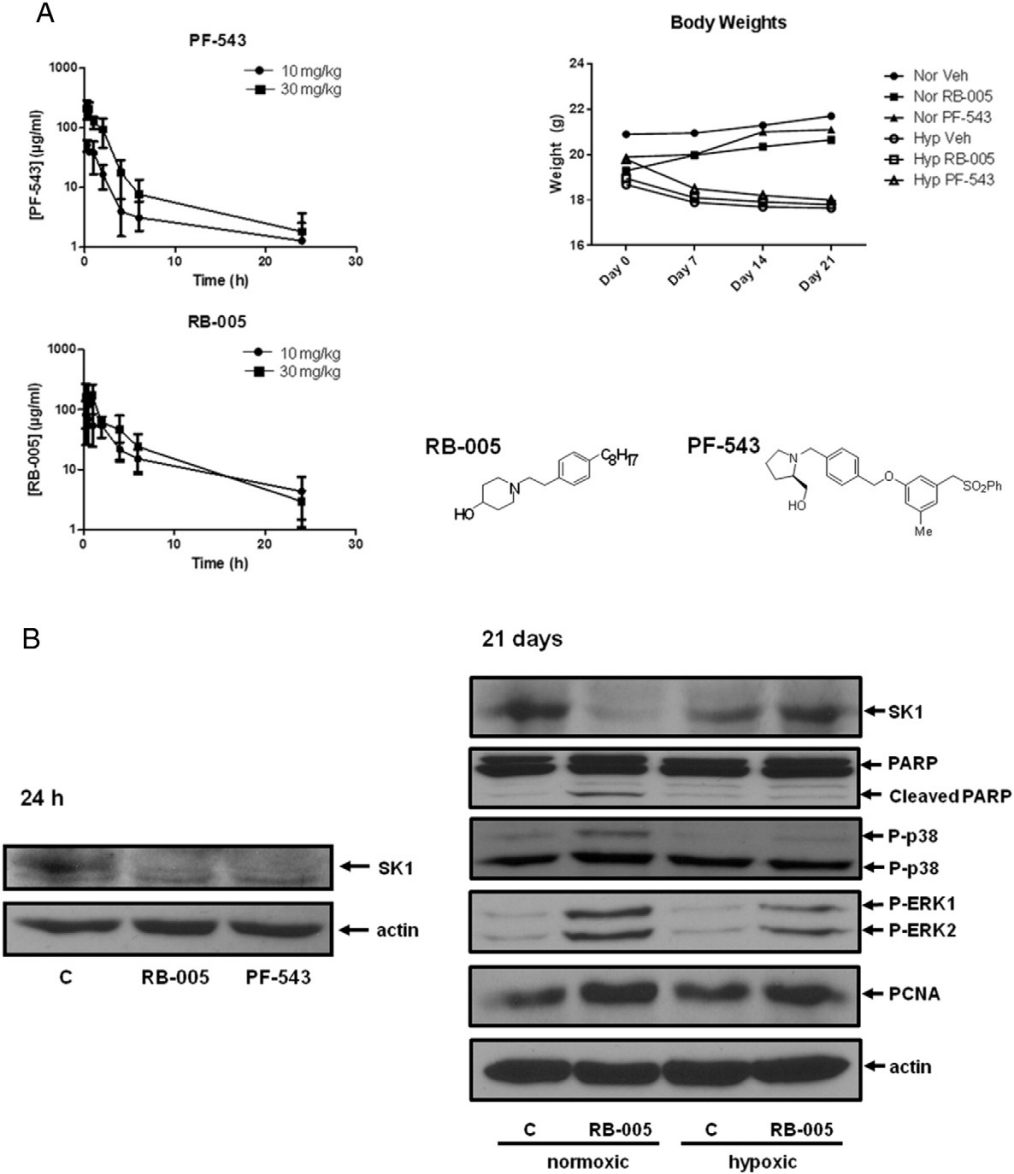

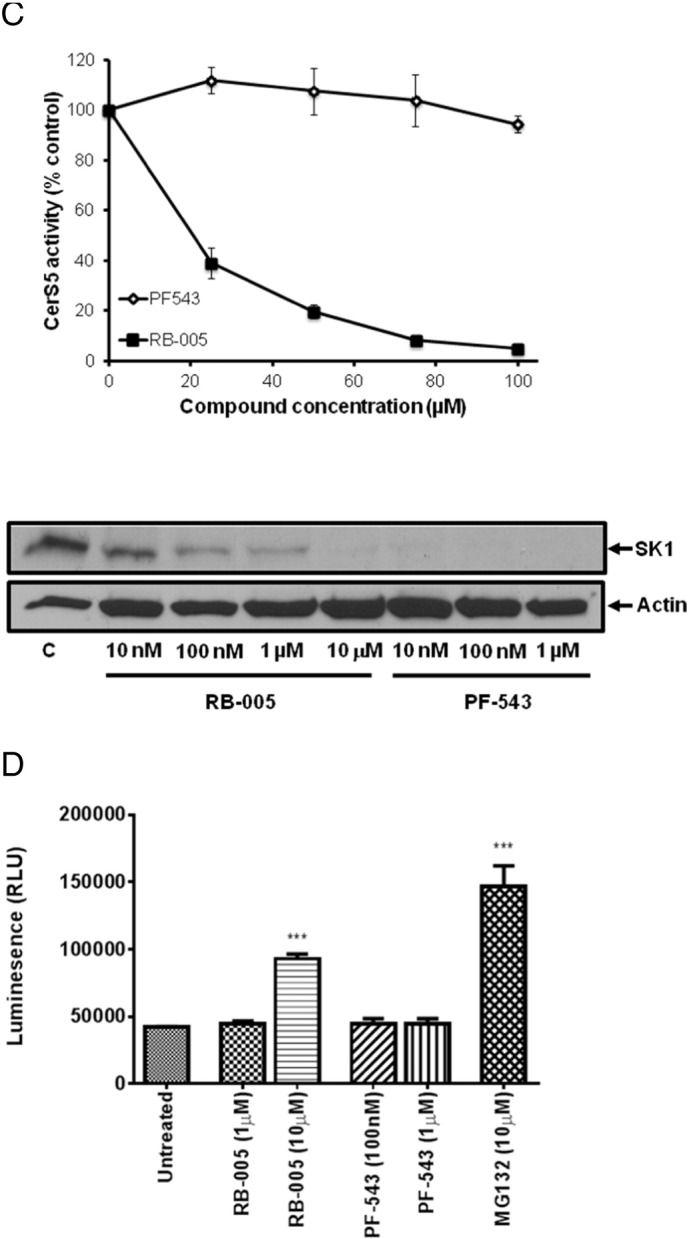
Effect of PF-543 and RB-005 on body weight, blood concentration, SK1 activity and expression in pulmonary arteries and cultured human pulmonary arterial smooth muscle cells**.** (A) Pharmacokinetics of RB-005 (10 and 30 mg/kg) and PF-543 (10 and 30 mg/kg) during 24 h treatment with these compounds. Also shown is the body weight of mice exposed to hypoxia (for 21 days) in the presence and absence of RB-005 (10 mg/kg) or PF-543 (1 mg/kg). n = 4–6 mice/group (results are expressed as mean ± SD). The chemical structure of PF-543 and RB-005 is shown. (B) Western blot of biomarkers in pulmonary vessels from mice exposed to RB-005 (10 mg/kg) or PF-543 (10 mg/kg) for 24 h (left panels) or hypoxia (for 21 days) in the presence and absence of RB-005 (10 mg/kg) (Right panels). Actin was used as a protein loading control. Blots from acute (24 h) treatments are of pulmonary arteries pooled from 4 mice, representative of 2 independent experiments. Hypoxic study blots display pulmonary arteries pooled from 4 animals, representative of 2 or 3 independent experiments. (C) Effect of PF-543 or RB-005 on CerS5 activity (activity (pmol/min/mg protein) expressed as % of control and is mean ± SD, n = 3) and western blot showing the effect of PF-543 or RB-005 on SK1 expression in cultured PASMC (24 h treatment). Actin was used as a protein loading control. Western blot results are representative of three independent experiments. (D) Effect of PF-543 or RB-005 on caspase 3/7 activity in cultured human pulmonary arterial smooth muscle cells (24 h treatment). MG132 (proteasomal inhibitor) was used as a positive control. Results are expressed as mean caspase 3/7 activity ± SD for n = 3 experiments. *** p < 0.001 *versus* control.

Mice were initially dosed (ip) with 10 mg/kg or 30 mg/kg of PF-543 for 24 h and the T_0.5_ was 1.2 h in blood samples (Fig. 1A). We have previously shown that SK1 inhibitors can bind to SK1 to induce the proteasomal degradation of SK1 [22], [23] and this is a biomarker for target engagement. Indeed, administration of 10 mg/kg PF-543 for 24 h to mice induced a decrease in SK1 expression in pulmonary vessels (Fig. 1B). Moreover, treatment of cultured PASMC with PF-543 for 24 h abolished SK1 expression at nM concentrations which correlates with the K_i_ for inhibition of SK1 activity (Fig. 1C, [22]), indicating that *in vivo* effects of PF-543 are recapitulated in an *in vitro* pulmonary arterial smooth muscle cell model.

We calculated that 10 mg/kg ip dosing of PF-543 yields an initial blood concentration of 50 μg/mL blood, which corresponds to ~ 100 μM. We therefore reduced the dosing of PF-543 to 1 mg/kg for chronic exposure experiments, a dose that has previously been shown to be sufficient for SK1 inhibition and reduction of serum and cardiac S1P levels in mice [19]. Chronic hypoxia prevented weight gain but PF-543 had no effect over hypoxia (Fig. 1A). We also used the SK1 selective inhibitor, RB-005 [23]. In contrast with PF-543, RB-005 is also an inhibitor of ceramide synthase (Fig. 1C). This has allowed us to investigate the effect of modulating ceramide biosynthesis in regulating vascular remodelling in the hypoxic PAH model. The IC_50_ for inhibition of SK1 is 3.6 μM [23] and for ceramide synthase 5 it was ~ 20 μM (Fig. 1C). RB-005 is an FTY720 analogue and indeed, FTY720 has also been shown to also inhibit ceramide synthase; dependent on the chain length of the acyl CoA substrate [28]. Paradoxically, FYT720 increased ceramide levels in HEK cells [28], which might be due to either kinetic considerations [28] and/or due to the fact that FTY720 is also an inhibitor of SK1 [21].

At 10 mg/kg or 30 mg/kg of RB-005, the T_0.5_ was 2.1 h in blood samples (Fig. 1A). There was no adverse effect on body weight in animals chronically dosed with RB-005 for 21 days (Fig. 1A). Administration of RB-005 (10 mg/kg) (which corresponds to ~ 30 μM initially, assuming 100% bioavailability) *in vivo* for 24 h also reduced SK1 expression in pulmonary vessels under normoxic conditions (Fig. 1B). This was confirmed using cultured PASMC where RB-005 reduced SK1 expression (Fig. 1C, [23]). Administration of RB-005 (10 mg/kg) to mice for 21 days reduced SK1 expression *in vivo* in pulmonary vessels from mice maintained under normoxic conditions, but had no effect under hypoxic conditions (Fig. 1B). The failure of RB-005 to reduce SK1 expression in blood vessels under hypoxic conditions might suggest resistance of SK1 to proteasomal degradation. Indeed, a similar resistance to SK1 inhibitors has been observed in certain cancer cells [29]. The mechanism is unknown but suggests that a possible hypoxic-dependent post-translational modification of SK1 might increase its stability *in vivo*.

RB-005 induced an increase in PARP cleavage and the phosphorylation of ERK-1/2 and p38 MAPK in vessels from mice maintained under normoxic conditions suggesting that this inhibitor might promote vascular injury itself (Fig.1B). These responses were absent or diminished after exposure to chronic hypoxia (Fig. 1B). PCNA expression was also increased by RB-005 under both normoxic and hypoxic conditions (Fig. 1B). RB-005 (10 μM) exhibits a different mechanism of action compared with PF-543, as it induced caspase-3/7 activity in cultured human pulmonary smooth muscle cells (Fig. 1D). These findings suggest that inhibition of ceramide synthase and SK1 activity might be the most effective means of inducing apoptosis of human pulmonary smooth muscle cells *in vitro* under normoxic conditions.

### 3.2 Effect of RB-005 or PF-543 on right ventricular pressure and pulmonary vascular remodelling

**Fig. 2:**
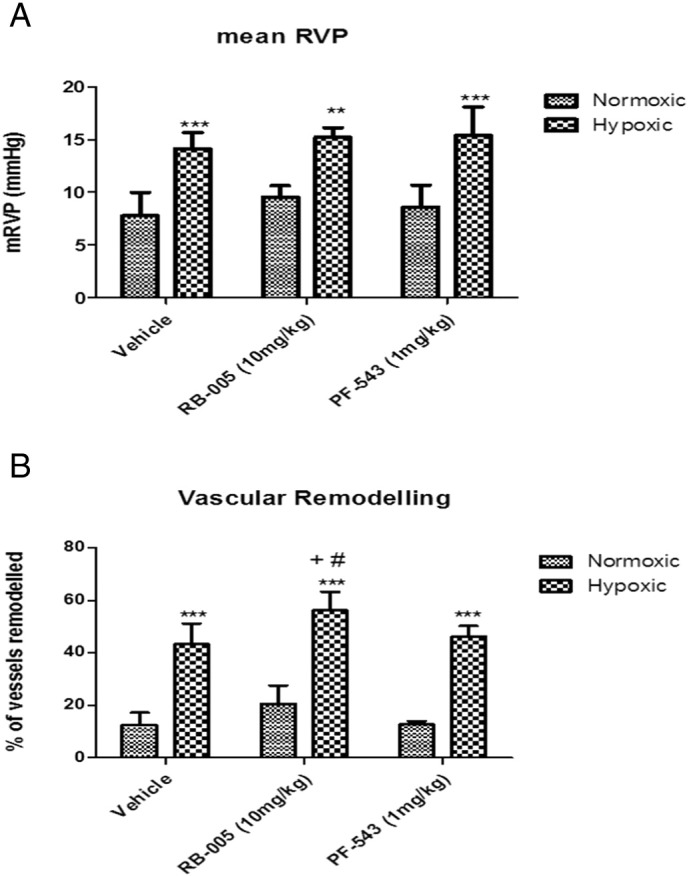
Effect of PF-543 and RB-005 on RVP and vascular remodelling. Effect of RB-005 (10 mg/kg) or PF-543 (1 mg/kg) on (A) RVP (vehicle n = 7–8 mice, RB-005 n = 3–5 mice/group; PF-543, n = 3–4 mice/group, ** p < 0.01, *** p < 0.001 *versus* normoxic) and (B) vascular remodelling (n = 4–6 mice per group, *** p < 0.001 *versus* normoxic; + p < 0.01 *versus* hypoxic vehicle; # p < 0.05 *versus* PF-543/hypoxic).

Mice subjected to 21 days of hypoxia exhibited a marked increase in mean right ventricular pressure (RVP) compared to normoxic controls (Fig. 2A) and this was associated with a significant increase in vascular remodelling (Fig. 2B). We noted that hypoxia did not increase phosphorylated ERK-1/2 levels in the arteries (Fig. 1B). The effect of hypoxia on PASMC proliferation is controversial, particularly under cell culture conditions with both growth promoting and suppressing phenotypes being observed [30]. In whole artery preparations, such as those used here, the dominant cell type proliferating under hypoxic conditions is likely to be pulmonary artery fibroblasts rather than PASMCs (which undergo hypertrophy rather than hyperplasia) which proliferate in an ERK1/2 independent manner [31].

**Fig. 3:**
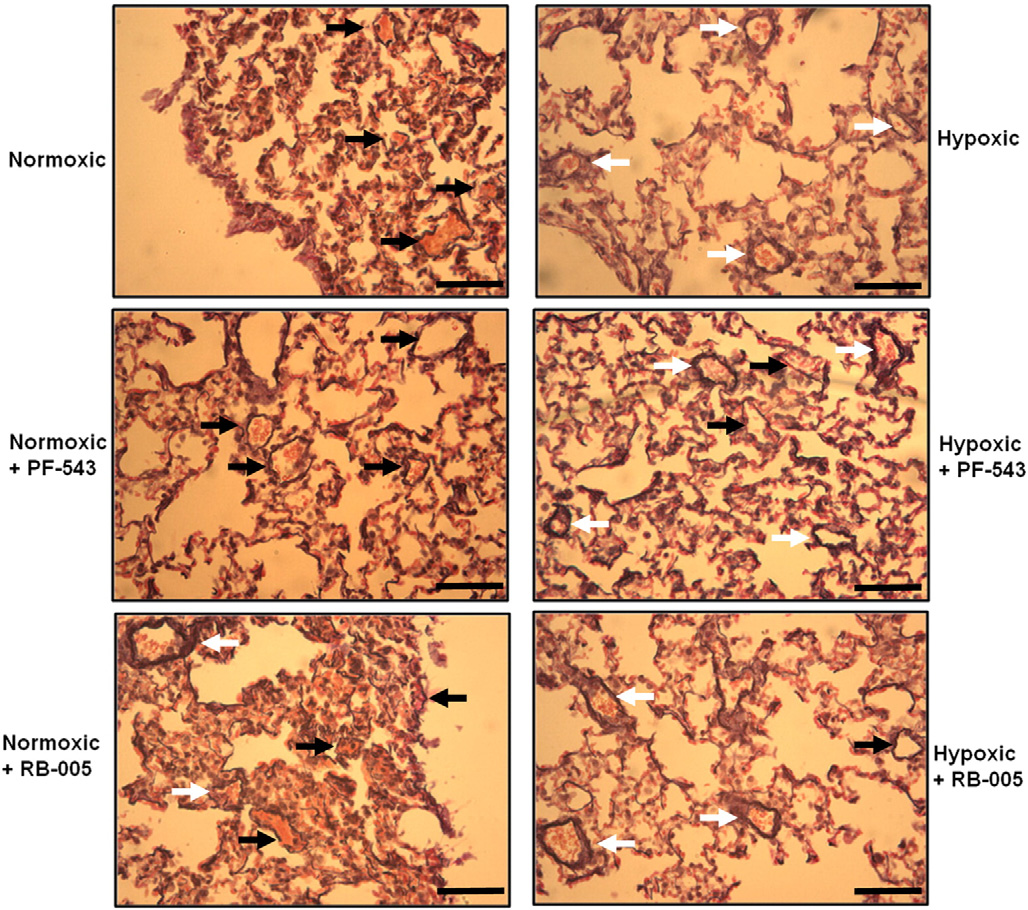
Representative photomicrographs of pulmonary vascular remodelling. Effect of RB-005 (10 mg/kg) or PF-543 (1 mg/kg) on vascular remodelling. Images are representative of 4–6 mice/group. Scale bar represents 100 μm. Black arrows indicate non-remodelled vessels while white arrows indicate remodelled vessels.

Administration of 1 mg/kg PF-543 or 10 mg/kg RB-005 at the onset of hypoxia had no effect on mean RVP (Fig. 2A). While PF-543 had no effect (Figs. 2B, 3), RB-005 modestly enhanced hypoxia-dependent vascular remodelling (Figs. 2B, 3). This enhancement was evident in terms of absolute number of remodelled vessels and also in more extensive elastin staining. These findings are associated with a failure of RB-005 to reduce SK1 expression in vessels from mice treated with hypoxia for 21 days (Fig. 1B) and an RB-005-dependent increase in the level of phosphorylated ERK-1/2 and p38 MAPK, which could contribute to a proliferative response. Most significantly, RB-005 failed to induce PARP cleavage in vessels from mice that had been subjected to hypoxia for 21 days (Fig. 1B) and this might reflect a reduced apoptotic rate that could account for the increase in vascular remodelling in response to RB-005. This is also consistent with the possibility that the inhibition of ceramide synthase by RB-005 might reduce the formation of apoptotic ceramide.

### 3.3 Biochemical markers associated with right ventricular hypertrophy

**Fig. 4:**
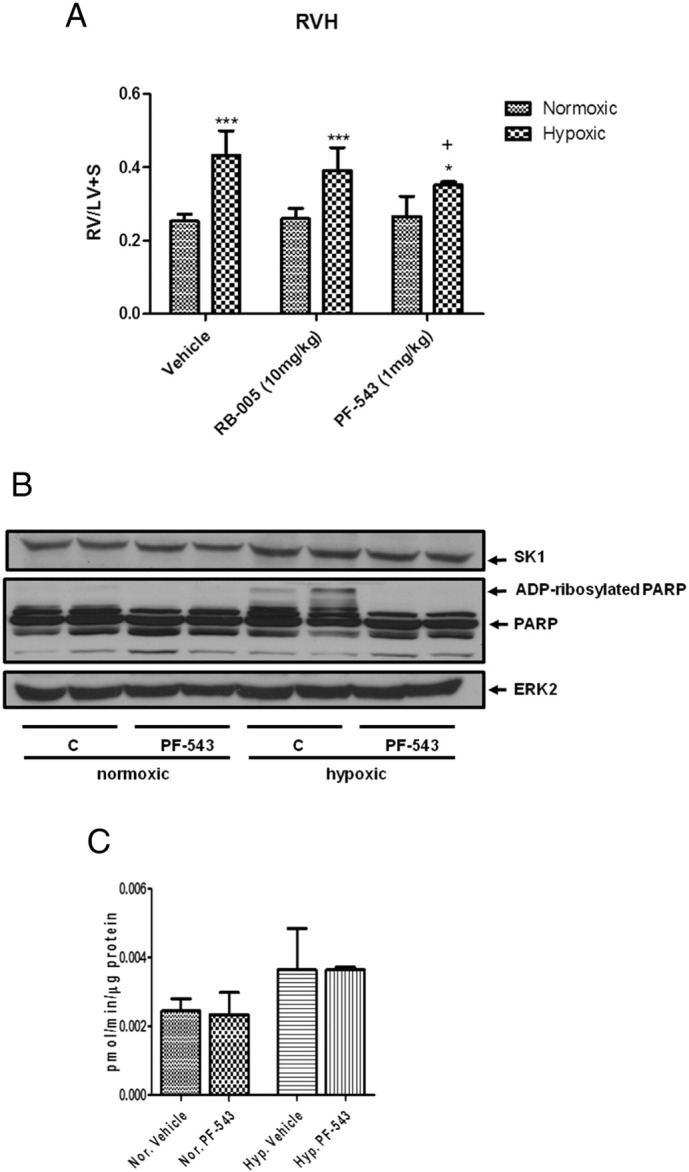

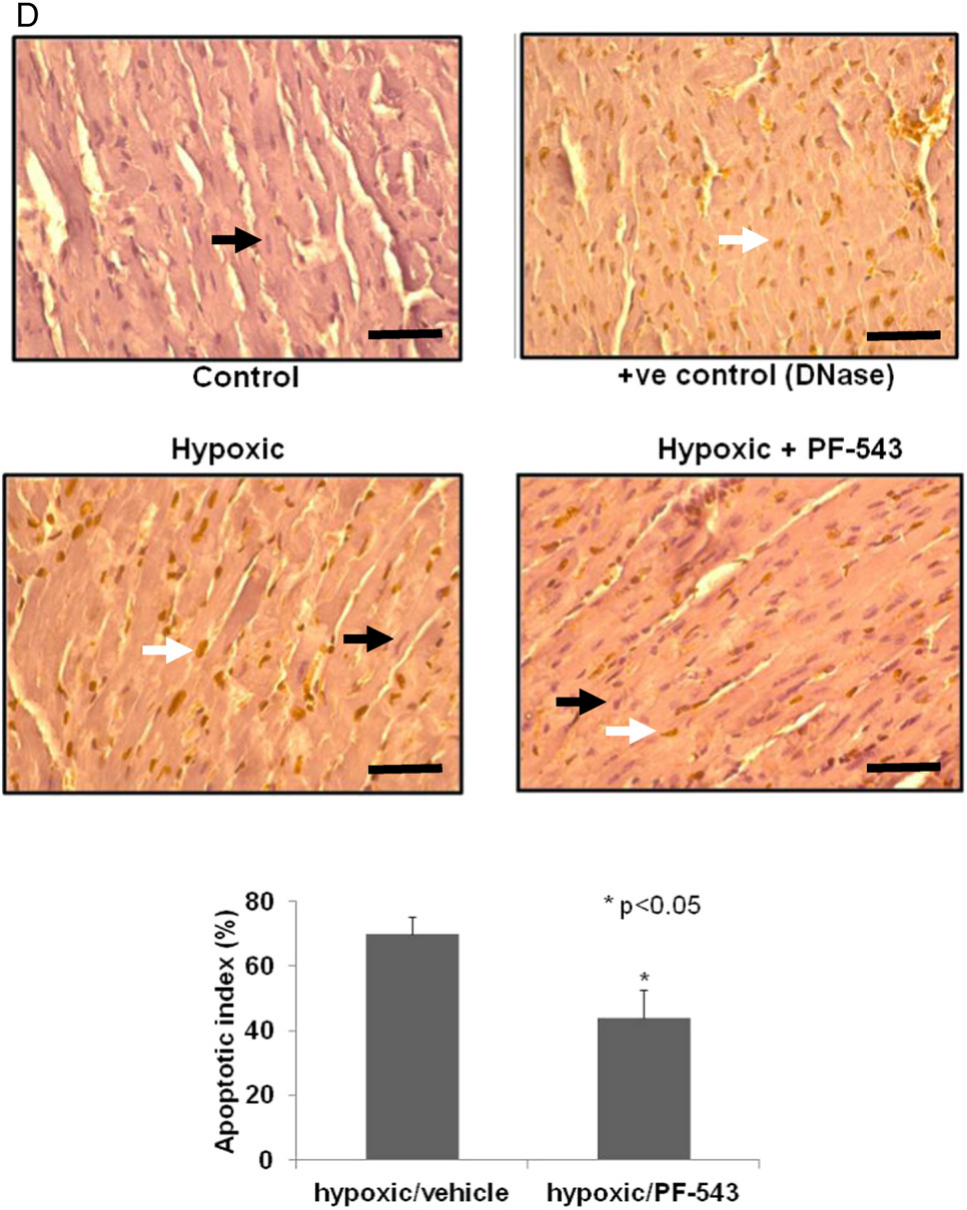
Effect of PF-543 and RB-005 on right ventricular hypertrophy. (A) Effect of RB-005 (10 mg/kg) or PF-543 (1 mg/kg) on right ventricular hypertrophy (n = 4–10 mice/group, * p < 0.05, *** p < 0.001 *versus* normoxic; + p < 0.05 *versus* vehicle/hypoxic). (B) Western blot of PARP processing in right ventricles of mice exposed to normoxia or hypoxia and vehicle or PF-543 (1 mg/kg) for 21 days. ERK-2 was used as a protein loading control. Results are representative of n = 6 mice/group (each lane corresponds to a separate animal). (C) Right ventricular SK1 activity. Results are expressed as mean activity (pmol/min/μg protein) ± SD (n = 3 mice). (D) Images of TUNEL staining in right ventricles from hypoxic and PF-543/hypoxic treated mice (3 mice/group) with quantification of apoptotic index. DNase was used as a positive control. Scale bar represents 50 μm. Black arrows indicate negative TUNEL staining of cardiomyoctes, while white arrows indicate positive TUNEL (brown colour) staining of cardiomyocytes.

Interestingly, PF-543 but not RB-005 significantly reduced right ventricular hypertrophy (RVH) (Fig. 4A). It is frequently assumed that increased RVH is subsequent to and correlates with elevated RVP yet data from animal models has cast doubt on this assumption with sustained RVP existing in the absence of significant RVH [31]. Conversely, RVH has been observed to develop in mice lacking peripheral serotonin following chronic exposure to hypoxia despite RVP remaining normal [32]. This is suggestive of a direct effect of hypoxia on cardiac hypertrophic signalling pathways. Therefore, our findings with PF-543 do not support a major protective function of SK1 on vascular remodelling, but instead invoke a direct role in cardiac dysfunctional hypertrophy. The failure of the SK1 inhibitor, PF-543, to reduce vascular remodelling contrasts with finding that *Sk1*^−/−^ mice are protected from hypoxic-induced pulmonary hypertension [8]. However, PF-543 administration is for 21 days and does not reflect the same chronic system in which the *Sk1* gene is deleted. In addition, SK2 might compensate for the loss of SK1 in *Sk1* knockout mice and this is clearly evident from the finding that *Sk1* or *Sk2* knockout mice survive, while dual *Sk1/Sk2* knockout mice do not survive, suggesting some redundancy in the function of the two kinases [33], [34]. Chen et al., [8] have also demonstrated a protective effect of the dual SK1/SK2 inhibitor, SKi (2-(*p*-hydroxyanilino)-4-(*p*-chlorophenyl)thiazole). However, SKi also inhibits dihydroceramide desaturase [35] and the effect on vascular remodelling is unknown.

To establish the mechanism by which PF-543 is protective against RVH, we measured the expression level of several proteins that are known to be involved in the development of hypertrophy. First, we found that neither hypoxia nor PF-543 had any effect on right ventricular SK1 expression (Fig. 4B) or SK1 activity (Fig. 4C). The results concerning SK1 activity confirm the western blot analysis which showed no changes in SK1 expression in response to PF-543 in right ventricle homogenates. We also found that hypoxia induces the appearance of ADP-ribosylated PARP (M_r_ > 120 kDa) in the right ventricular tissue and this is completely blocked by administration of PF-543 to mice (Fig. 4B). ADP ribosylation of PARP is an early step involved in its proteolytic degradation preceding apoptosis [36]. We therefore used TUNEL staining (to detect double stranded DNA nicks that are a marker of apoptosis/necrosis) on right ventricular sections. Significant positive TUNEL staining was observed in the right ventricular sections from hypoxic treated mice (Fig. 4D), indicating significant apoptosis/necrosis of cardiomyocytes is induced by hypoxia and suggesting deleterious progression of right ventricular phenotype. Administration of PF-543 (1 mg/kg) to the mice caused a ~ 50% reduction in TUNEL staining (Fig. 4D) indicative of significant protection against apoptosis/necrosis and consistent with the reduction in RVH.

### 3.4 Effect of PF-543 on signalling biomarkers of hypertrophy

**Fig. 5:**
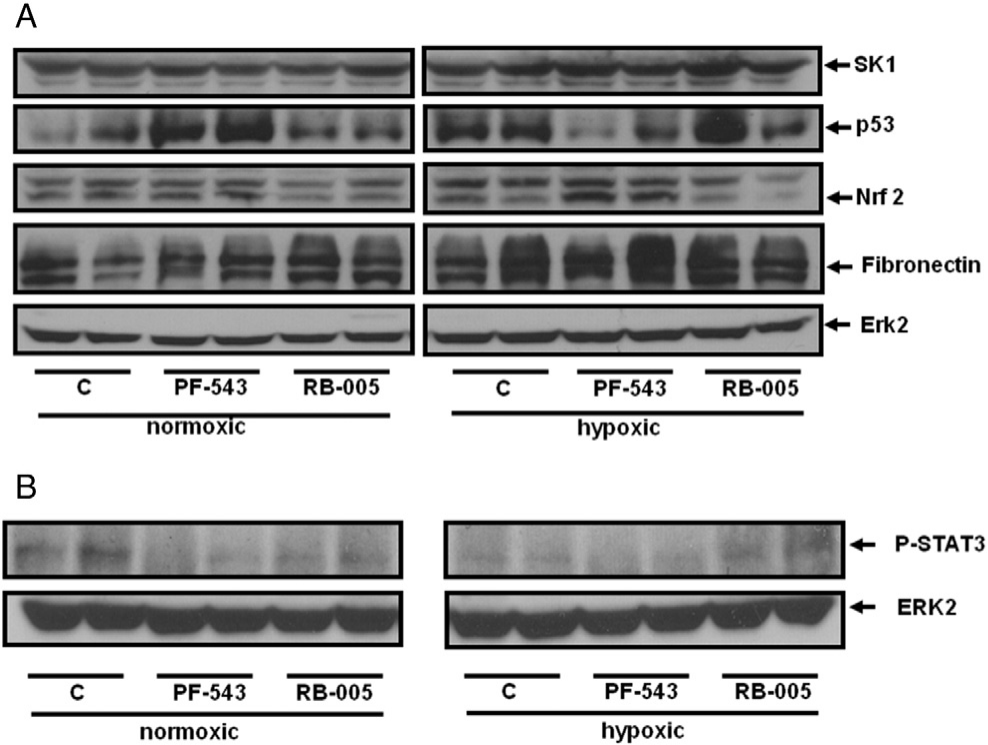
Effect of PF-543 and RB-005 on biomarkers in right ventricular hypertrophy. Western blot of biomarkers (A) p53, Nrf-2, SK1, fibronectin (results are representative of 3–5 mice and each lane corresponds to a separate animal); (B) P-STAT3 (results are representative from n = 2 mice/group and each lane corresponds to a separate animal) in right ventricles from mice exposed to RB-005 (10 mg/kg) or PF-543 (1 mg/kg) under normoxic or hypoxia (21 days) conditions. ERK-2 was used as a protein loading control.

The protection afforded by PF-543 (1 mg/kg) in preventing cardiomyocyte apoptosis/necrosis in right ventricles of mice exposed to hypoxia involves a reduction in the expression of p53 (that promotes cardiomyocyte death) (Fig. 5A) and an increase in the expression of an anti-oxidant protein, nuclear factor (erythroid-derived 2)-like 2 (Nrf-2) (Fig. 5A). Nrf-2 is a potentially key molecule in preserving right ventricular function, which might be up-regulated in an attempt to limit damage to the RV. Indeed, a detailed investigation of the RV in rats subjected to a severe angioproliferative model of PAH that results in RV failure revealed RV fibrosis, capillary loss and cardiomyocyte apoptosis, which could be partially reversed with Nrf-2 antioxidant treatment suggesting oxidative damage may contribute to the failing RV [37]. PF-543 (1 mg/kg) had no effect on fibronectin expression (Fig. 5A) indicating that fibrosis is not affected in early stage PAH. Interestingly, PF-543 increased p53 expression and had no effect on Nrf-2 expression under normoxic conditions (Fig. 5A), suggesting that its effect on hypertrophy under hypoxic conditions involves targeting the hypoxic-dependent and disease-forming mechanism. RB-005 (10 mg/kg) had no effect on the hypoxic-dependent regulation of p53, Nrf-2 or fibronectin either under hypoxic or normoxic conditions (Fig. 5A).

Cardiac SK1/S1P/S1P_1_ signalling has recently been shown to promote chronic cardiac inflammation and cardiac remodelling and dysfunction post-MI [19]. In this regard, PF-543 (1 mg/kg) administration to mice caused a reduction in phosphorylated STAT3 levels in the right ventricles from both normoxic and hypoxic mice. While RB-005 (10 mg/kg) reduced STAT3 phosphorylation under normoxic conditions, it had a less marked effect (and might conceivably stimulate STAT3 phosphorylation) under hypoxic conditions (Fig. 5B). Hypoxia also reduced STAT3 phosphorylation levels (Fig. 5B). These findings suggest that the changes observed with PF-543 on hypertrophy are not specific to the hypoxic-dependent changes in the STAT3 inflammatory pathway. Therefore, we did not find support for the model proposed by Zhang et al., [19], at least in the PAH model.

## 4 Conclusion

**Fig. 6:**
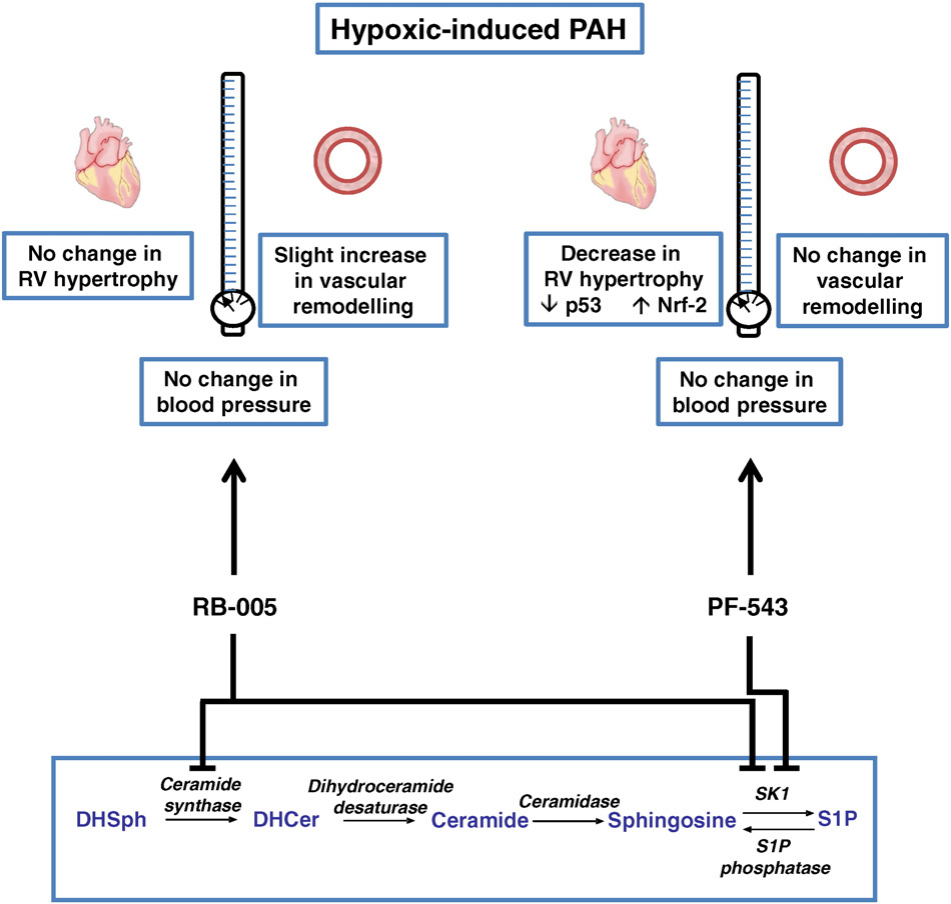
Summary of the effects of PF-543 and RB-005 on RVP, vascular remodelling and right ventricular hypertrophy in a hypoxia treated mouse model. Schematic shows that SK1 inhibitor, PF-543 reduces right ventricular hypertrophy without affecting vascular remodelling and RVP under hypoxic conditions. This is due to protection against cardiomyocyte apoptosis and is associated with a reduction in p53 expression and an increase in anti-oxidant Nrf-2 expression. In contrast, RB-005, which inhibits both ceramide synthase and SK1 has no effect on right ventricular hypertrophy or RVP and slightly increased vascular remodelling under hypoxic conditions suggesting that inhibition of ceramide synthase might negate the effect of inhibiting SK1 in PAH.

We have identified a compound, PF-543 (a potent sphingosine kinase 1 inhibitor) which has a dramatic effect on hypertrophic-induced myocardial apoptosis, which is the major killer in humans suffering from PAH. The findings of the current study demonstrate that PF-543 failed to reduce vascular remodelling. However, PF-543 did reduce dysfunctional hypertrophy, associated with protection against cardiomyocyte apoptosis (Fig. 6). This protection against cardiomyocyte apoptosis occurs as the development of dysfunctional hypertrophy is reduced and therefore fewer cardiomyocytes reach the stage where they are prone to apoptosis. These effects are not recapitulated by RB-005, suggesting that inhibition of ceramide synthase negates the beneficial effects of inhibiting SK1 activity on hypertrophy in PAH (Fig. 6). Therefore, it will be necessary to produce compounds that lack inhibitory activity against ceramide synthase (active in both the *de novo* ceramide synthesis pathway and also in the sphingolipid rheostat) in order to execute the effect of inhibiting SK1 on hypertrophy in PAH.
